# Biological Processes that Prepare Mammalian Spermatozoa to Interact with an Egg and Fertilize It

**DOI:** 10.6064/2012/607427

**Published:** 2012-05-29

**Authors:** Daulat R. P. Tulsiani, Aïda Abou-Haila

**Affiliations:** ^1^Department of Obstetrics and Gynecology, Vanderbilt University School of Medicine, Nashville, TN 37232, USA; ^2^UFR Biomédicale, Université Paris Descartes, 75270 Paris Cedex 06, France

## Abstract

In the mouse and other mammals studied, including man, ejaculated spermatozoa cannot immediately fertilize an egg. They require a certain period of residence in the female genital tract to become functionally competent cells. As spermatozoa traverse through the female genital tract, they undergo multiple biochemical and physiological changes collectively referred to as capacitation. Only capacitated spermatozoa interact with the extracellular egg coat, the zona pellucida. The tight irreversible binding of the opposite gametes triggers a Ca^2+^-dependent signal transduction cascade. The net result is the fusion of the sperm plasma membrane and the underlying outer acrosomal membrane at multiple sites that causes the release of acrosomal contents at the site of sperm-egg adhesion. The hydrolytic action of the acrosomal enzymes released, along with the hyperactivated beat pattern of the bound spermatozoon, is important factor that directs the sperm to penetrate the egg coat and fertilize the egg. The sperm capacitation and the induction of the acrosomal reaction are Ca^2+^-dependent signaling events that have been of wide interest to reproductive biologists for over half a century. In this paper, we intend to discuss data from this and other laboratories that highlight the biological processes which prepare spermatozoa to interact with an egg and fertilize it.

## 1. Introduction

Mammalian fertilization, a species-specific event, is the net result of a highly orchestrated process by which two radically different looking cells, sperm and egg, interact and unite to form a zygote, a cell with somatic chromosome numbers. The tight and irreversible binding of the opposite gametes in the mouse and other mammals studied, including man, starts a calcium- (Ca^2+^-) dependent signal transduction pathway that results in the exocytosis of acrosomal contents at the site of sperm binding [1, 2]. The hydrolytic action of the acrosomal glycohydrolases and proteinases, released at the sperm binding site, along with the enhanced thrust generated by the hyperactivated spermatozoon are important factors that regulates the penetration of the zona pellucida (ZP), the extracellular glycocalyx that surrounds the egg, and fertilize it [2, 3].

Accumulated evidence from several laboratories has suggested that sperm surface receptor(s) recognize and bind to glycan units on ZP3, one of the 3-4 glycoproteins that form the ZP in different species, in a species-specific manner (for reviews see [2, 4, 5]). The glycan chains of many glycoproteins are implicated in cell-cell interactions, including sperm-egg adhesion [2, 6]. Several sperm surface molecules have been suggested to function as receptors that recognize terminal sugar residues on ZP3 [2, 5]. The sugar residues suggested to be recognized by the capacitated spermatozoa in various species include mannosyl [7–9], sialyl [10], glucosaminyl [11], and galactosyl [12]. Although a terminal fucosyl residue has not been implicated in sperm binding, its presence has been suggested to be obligatory for an oligosaccharide to bind spermatozoa with high affinity [13].

In spite of the overwhelming evidence implicating that sperm-egg adhesion is a carbohydrate-mediated event (see above), Dr. Dean and associates recently tested a mouse ZP2 cleavage model and a glycan release model for the recognition of the opposite gametes. Data from these studies provided evidence suggesting that sperm-egg recognition, at least in the mouse, depends on the cleavage status of the ZP2 [14]. Until and unless additional evidence is available, it is reasonable to suggest that the precise cause for the sperm-egg adhesion is far from being resolved.

There has been a long-standing interest in the basic biology of sperm-egg adhesion and fertilization process. Our success of *in vitro* fertilization in domestic animals and humans is a result of the knowledge of these events gained in animal models. Various events that lead to sperm-egg adhesion and fertilization of the egg are best understood in the mouse, although there is some information in other mammals. Successful fertilization in the mouse involves several well-orchestrated events. They are (1) changes in spermatozoa during their formation and development in the testes; (2) sperm maturation in the epididymis, (3) sperm capacitation in the female reproductive tract, (4) adhesion of capacitated spermatozoa to the ZP, (5) exocytosis of the acrosomal contents (i.e., induction of the acrosome reaction (AR)), and (6) secondary binding events that lead to sperm-egg fusion. We will highlight these events and discuss their importance in the process of fertilization. Our intention is also to discuss signal transduction pathways that regulate sperm capacitation and the AR, two prerequisite events before sperm can fertilize an egg. 

## 2. Formation and Development of Spermatozoa in the Testes

### 2.1. Formation of Spermatozoa

Throughout postpubertal male reproductive life, spermatozoa are formed from spermatogonial cells by a complex process referred to as spermatogenesis. In the sexually mature male, the two testicles or the testes produce and store millions of tiny microscopic structures, the spermatozoa. The entire process of sperm formation consists of three sequential phases of cell proliferation and differentiation [15–17]. First, there is an extensive multiplication and proliferation of spermatogonial cells to produce an optimal number of spermatogonia that give rise to early (preleptotene, leptotene, and zygotene) and late (pachytene) primary spermatocytes and also to maintain a pool of stem cells. Second, each primary spermatocyte undergoes a lengthy meiotic cell division that results in the formation of two secondary spermatocytes. Third, each secondary spermatocyte undergoes the second meiotic cell division to produce two haploid round spermatids. The round spermatids are incapable of further division. These cells have spherical nucleus with dispersed chromatin and the absence of nucleoli. By a complex set of nuclear and cytoplasmic changes, the ordinary-looking round spermatids gradually undergo remodeling of the nuclear and cellular components during their transformation into spermatozoa by a process referred to as spermiogenesis [15]. The net result is the formation of hydrodynamically shaped spermatozoa containing two parts: (i) the head with a nucleus and an acrosome (anterior head) and a postacrosomal region and (ii) the flagellum comprising of the middle, principal, and end pieces. Many details of spermiogenesis have been described previously [17] and will not be repeated in this paper.

Sperm development takes place within the germinal epithelium of seminiferous tubules in the testes where spermatogenic cells are arranged along the basal membrane in a well-defined combination of various developmental stages. Due to the continuous maturation of the testicular germ cells, the combination of cells varies along the length of the tubule. The well-coordinated multiple changes that occur between the appearance of one pattern and its disappearance in a different segment of a given tubule are defined as, “the cycle of seminiferous epithelium.” The entire process of spermatogenesis is dependent on the specific environment provided by the somatic (Sertoli and Leydig) cells in the testes and requires endocrine and paracrine as well as cell-cell interactions [17].

### 2.2. The Development of Sperm Cells during Spermatogenesis

Despite many advances in our understanding of various phases during spermatogenesis and spermiogenesis, little is known about the events that regulate the formation of spermatozoa. Multiple biochemical and histochemical approaches have provided evidence for the presence of testis-specific genes [18] as well as several cell surface antigens on pachytene spermatocytes [19]. Additional cell surface antigens are first detected on round spermatids following meiotic reduction division [15, 16]. Combined, these studies suggest that the stage-specific appearance of the cell surface molecules on diploid and haploid cells are important events in the development of spermatozoa. 

In addition to the antigens that are synthesized by the developing spermatogenic cells and incorporated into the sperm membranes, there is evidence that these molecules undergo modifications during spermatogenesis. Three mechanisms have been recognized: (i) elimination or masking of antigens, (ii) modification of glycan units of the existing glycoproteins, and (iii) selective proteolysis of the existing antigens, a process that converts enzymatically inactive precursor form into an enzymatically active mature form [20, 21]. Many details of sperm formation have been described in earlier articles [6, 17]. However, we will like to emphasize that, although the finally formed spermatozoa released from the seminiferous tubules (testicular spermatozoa) are morphologically differentiated cells, they are neither motile nor able to fertilize an egg. These functions develop over a period of time in the epididymis, a process referred to as epididymal maturation of spermatozoa (see below).

## 3. Sperm Maturation in the Epididymis

Along each testicle is an epididymis and vas deferens (vas) that make up the network of the male reproductive system. The epididymides are a set of two coiled tubes, one from each testicle, where testicular spermatozoa undergo many biochemical and morphological changes collectively called epididymal maturation [22–26]. The convoluted tubules are connected to vas, a pair of muscular tubes that transport sperm-containing fluid to uretha and semen out of the body through penis.

As stated above, the testicular spermatozoa are morphologically differentiated cells. However, they are neither motile nor capable of fertilizing an egg. These properties develop over a period of time as spermatozoa traverse through the epididymis. The tubule is divided into three regions: proximal (caput), middle (corpus), and distal (cauda). The precise region of the epididymis where the sperm cells undergo most of the modifications remains evasive. However, the caudal spermatozoa are motile and fertilization-competent cells. During epididymal passage, spermatozoa interact with epididymal luminal secretions. The epithelial cells lining the epididymal duct form a luminal fluid environment by actively secreting and absorbing small molecules (sugars electrolytes, etc.) and macromolecules (proteins, glycoproteins, etc.). Thus epididymal duct secretions mixed with the testicular content, provide a specific environment in which functionally immature spermatozoa undergo multiple modifications [22, 24, 25]. The net result is the production of the self-propelled and functionally competent spermatozoa. 

It is important to emphasize that spermatozoa are surrounded by the plasma membrane (PM) that mediates many of the early events leading to sperm-egg adhesion and fertilization. The sperm PM components are first expressed in the diploid cells and are continuously modified during sperm development in the testes and maturation in the epididymis. Several sperm surface molecules are synthesized in a high-molecular-weight precursor form that undergoes site-specific proteolytic processing in the testis and epididymis. Many details of these molecules have been described in an earlier article [14]. 

It should be noted that glycan moieties of the sperm PM glycoproteins are extensively modified as spermatozoa transit from the caput to the cauda region. Since the spermatozoa entering the epididymal region have no known synthetic activities, the appearance of new molecules on the sperm PM and changes in the sperm function are thought to be due to their interaction with the luminal fluid within the epididymis. The above-mentioned changes in the sperm PM antigen(s) can be explained in a number of ways: (1) adsorption/association of the macromolecules from the luminal fluid to the sperm PM, (2) exposure of previously masked glycan chains of the existing glycoproteins, and (3) modification of preexisting glycoproteins by the action of glycohydrolases, glycosyltransferases and/or proteinases.

One such molecule is a 135–150 kDa glycoconjugate that is lost from the caput spermatozoa as the cells reach the caudal region [27, 28]. The apparent loss of the antigen could be due to the modification of the glycan chains (Gal-*β*-1,3-GalNAc-linkage) found in O-linked glycoproteins by the cleavage of the terminal galactosyl residue(s) by the luminal fluid *β*-D-galactosidases [29, 30] or the masking of the galactosyl residue(s) with the aid of luminal fluid glycosyltransferases [31]. How these two sets of enzymes may function in the epididymal luminal fluid environment has been discussed in a previous article [32].

In addition to the alteration of the sperm surface glycoproteins, there is evidence that sperm PM undergoes extensive remodeling and redistribution of antigens at various stages of sperm maturation in the epididymis. In guinea pig, fertilin is synthesized during spermatogenesis as a heterodimer containing *α*- and *β*-subunits. The *α*-subunit is processed during spermatogenesis whereas the processing of the *β*-subunit is delayed until sperm cells reach the distal caput region [33]. The net result is the reduction in the size of the protein dimer from 90 kDa to 80 kDa. Similarly, the boar sperm surface molecule, zonadhesin, a molecule homologous to both the von Willebrand factor and mucins, is first expressed in the testicular germ cells and is proteolytically processed to a heterodimer containing 105 kDa and 45 kDa subunits. To learn more about the processing, remodeling and redistribution of additional sperm surface molecules, interested readers should consult an earlier review article [34].

The maturation-associated modifications are not limited to the sperm surface molecules. Several intra-acrosomal components are modified during spermatogenesis and/or epididymal maturation. The growing list of these molecules include *β*-D-galactosidase [35], *α*-L-fucosidase [36], acrosin [37], SP-10 [38], and acrogranin [39]. Proacrosin-acrosin, a trypsin-like serine protease, is reported to be present in the sperm acrosome as well as on the sperm surface. The enzymatically inactive precursor (proacrosin) has been localized on the inner and outer acrosomal membranes as well as in the acrosomal matrix [40]. The large molecular precursor undergoes proteolytic processing in the spermatozoa from several species [37, 41, 42]. In guinea pig spermatozoa, the reduction in the size of proacrosin is reported to be due to the processing of its glycan moieties [39]. 

The above-mentioned and perhaps many more modifications on sperm surface and intra-acrosomal molecules during sperm maturation in the epididymis are essential before spermatozoa become functionally competent cells capable of undergoing capacitation in the female reproductive tract and fertilizing an egg (see below).

## 4. Capacitation: The Physiological Priming of Spermatozoa

In addition to the maturation in the epididymis, mammalian spermatozoa must undergo physiological priming (capacitation) between the events of mating and fertilization. At coitus, millions of spermatozoa are deposited in the female reproductive tract. A vast majority of the deposited male gametes are eliminated; however, a small percentage of spermatozoa rapidly enter the highly folded mucus-filled cervix that has three important roles. First, it prevents entry of seminal plasma into uterus. Second, it prevents the entry of morphologically abnormal spermatozoa as well as potentially infectious microbes into uterus. Finally, the cervix stores viable spermatozoa [1]. Once in the cervix, spermatozoa move by passive and active processes toward the uterus and oviduct, the likely site of *in vivo* fertilization in many species.

Spermatozoa that enter the distal oviduct region (ampulla), the site of fertilization, meet the ovulated egg(s) surrounded by cumulus complex. The complex is thought to be dispersed by hyaluronidase, an enzyme present within the sperm acrosome as well as the sperm surface, allowing capacitated and hyperactive spermatozoa free passage through the dispersed cumulus complex [3].

Much of the current knowledge on sperm capacitation has been provided by the pioneering studies by Austin [43] and Chang [44]. These two investigators independently demonstrated functional changes on spermatozoa recovered from the female genital tract after mating or preincubating the cauda epididymal spermatozoa with the oviductal secretions. The secretions collected from the oviduct of estrus females have been demonstrated to be most efficient in rendering the functional changes on spermatozoa.

Mammalian spermatozoa contain two parts: (1) a head with acrosomal (anterior head) and postacrosomal regions and (2) the flagellum comprising of the principal, mid, and end-pieces. Whereas the receptor molecule(s) that recognizes and binds to the zona-intact egg in a species-specific manner and undergoes the signal transduction pathway that induces the acrosomal reaction (AR) in many species (see below) is localized in the anterior head [3, 45], the hyperactive motility is a result of molecular changes in the flagellum [46]. Thus, capacitation is the net result of changes in the membrane properties and multiple enzyme activities in sperm head and flagellum that activate the cell signaling cascades before sperm-egg interaction and fertilization [1, 2].

All mammalian spermatozoa studied thus far undergo capacitation after residing in the female reproductive tract for a certain period of time. Spermatozoa can also be capacitated *in vitro* by incubating in a chemically defined medium supplemented with bovine serum albumin [47, 48] or methyl-*β*-cyclodextrin [49], energy substances, and reagents used in the Krebs-Ringer bicarbonate medium. Albumin, the major protein present in the female reproductive secretions, or methyl- *β*-cyclodextrin facilitates capacitation by efflux of sterols, mainly cholesterol, from the plasma membrane of the capacitating spermatozoa. The loss of cholesterol is thought to destabilize the sperm plasma membrane (PM) bi-layer making it permeable and fusogenic [1]. Experimental evidence suggests that there is a gradual loss of cholesterol during capacitation; however, spermatozoa do not undergo the external stimulus-induced AR for some time, a result consistent with the author's suggestion that cholesterol loss precedes capacitation [50]. In addition to the efflux of cholesterol, the capacitating spermatozoa undergo multiple biochemical changes including tyrosine phosphorylation of a subset of sperm molecules [46]. 

It should be noted that only capacitated spermatozoa can bind the zona-intact egg and undergo the AR, a result consistent with the suggestion that the anterior head region of these cells has undergone major changes. Interestingly, capacitating/capacitated spermatozoa have been demonstrated to undergo changes in the lectin-binding patterns [51]. Since lectins bind to the terminal sugar residues with high specificity and affinity, the observed changes in the lectin-binding patterns are indicative that the cell surface glycan units are altered in the anterior head region of capacitating/capacitated spermatozoa. Interestingly, we have demonstrated that the mouse spermatozoa incubated in a medium that favors capacitation undergo membrane priming. The net result is the exposure of intra-acrosomal glycohydrolases on the sperm surface in a time-dependent manner [52]. Many intra-acrosomal glycohydrolases are glycoproteins [3]. Their exposure on the cell surface of capacitating spermatozoa will be expected to alter their lectin-binding properties.

Yanagimachi and Chang [53] were the first to use the term hyperactivation to describe vigorous whiplash-like beating pattern of capacitated sperm flagellum. Hyperactivation has now been reported in all mammalian species examined and is often considered a change in flagellar motility adjacent to capacitation [54]. Thus, it is reasonable to conclude that, although the hyperactivated motility is associated with sperm capacitation, the two changes are regulated by independent mechanisms. Indeed, our group has demonstrated that it is possible to prevent *in vitro* capacitation with or without blocking the sperm motility [49]. Data presented in the paper have allowed us to suggest the occurrence of multiple signaling pathways that regulate sperm capacitation.

Accumulated evidence indicates that the flagellum of capacitating spermatozoa undergoes protein tyrosine phosphorylation in several species [55, 56], including human [57]. It is interesting to mention that the protein tyrosine phosphorylation has been implicated in the acquisition of the capacitation-associated hyperactive motility using multiple approaches. Combined, data presented in these articles are consistent with studies from our group [46] suggesting a relationship between capacitation-associated protein tyrosine phosphorylation and hyperactive sperm motility. The hyperactive beat pattern of the flagellum is thought to be necessary for spermatozoa to penetrate the cumulus complex surrounding the ovulated egg, bind to the zona-intact egg, and undergo the AR. The hydrolytic action of proteinases and glycohydrolases released at the site of sperm-zona (egg) binding, along with the enhanced thrust generated by the whiplash beat pattern of the bound spermatozoon, is important factor that regulates the penetration of the ZP, and fertilization of the egg [58]. 

It should be noted that, although several sperm molecules have been reported to be protein phosphorylated during capacitation and are thought to be important in regulating capacitation-associated events [49, 56], very few of these molecules have so far been characterized. Earlier investigators mostly used pharmacological approaches to examine their appearance during *in vitro* capacitation using anti-phosphotyrosine antibody. However, more recently, phosphoproteome analysis and gene-knockout approaches have provided more powerful tools to identify and characterize the proteins that are tyrosine phosphorylated during sperm capacitation. When these molecules will be characterized by these approaches, they will provide new insights on the physiological significance of protein tyrosine phosphorylation of a subset of sperm molecules during capacitation of male gametes.

Accumulated evidence indicates that Ca^2+^, HCO_3_^−^, and cAMP are the key secondary molecules that regulate sperm capacitation. For instance, bovine spermatozoa incubated under *in vitro* conditions that favor capacitation had 6-fold higher Ca^2+^ level. This increase in intrasperm Ca^2+^ will stimulate Ca^2+^-dependent ATPase and adenylyl cyclase, two key enzymes that participate in signaling cascade. Omission of extracellular Ca^2+^ from the medium blocks capacitation, but the protein tyrosine phosphorylation continues [46]. These data imply that the Ca^2+^ has a role in other events of capacitation. Calcium exerts its effect on sperm capacitation through Ca^2+^-binding proteins that undergo conformational changes upon interaction with the divalent cation. This interaction is thought to play an important role in several cell signaling pathways by modulating biological activities of multiple enzymes and ion pumps including Ca^2+^/calmodulin- (CaM-) dependent kinases [46]. 

It is important to emphasize that the presence of calcium in the capacitation medium is essential for maintaining motility of human spermatozoa. To determine the role of CaM-dependent kinase IV in the regulation of human sperm motility, Herr and associates used specific inhibitors of this kinase or their inactive analogue in the capacitation medium. Inclusion of inhibitors (but not the inactive analogues) in the capacitation medium resulted in progressive decrease in the motile spermatozoa without altering their viability, protein tyrosine phosphorylation, or follicular fluid-induced AR. The CaM-dependent kinase IV which increased during capacitation was immunolocalized to the sperm flagellum. Combined, these data demonstrate the presence and involvement of kinase IV in the regulation of sperm motility [59]. 

It is obvious from the above discussion that calcium is an important signaling molecule for sperm capacitation. The divalent cation exerts its effects on sperm capacitation through CaM and other Ca^2+^-binding proteins. Calmodulin, a 17 kDa Ca^2+^-binding acidic protein, is known to regulate many signaling events in somatic cells [60, 61]. Sperm cells contain high levels of CaM in the head and flagellum [62]; this localization is consistent with its reported role in sperm capacitation and induction of the AR [63]. Many details on the potential mechanism of the action of CaM on sperm function have been discussed in our articles [5, 49]. The experimental evidence presented in these articles has allowed us to suggest that CaM regulates sperm functions by modulating sperm membrane components.

Evidence accumulated over the years indicates that Ca^2+^ influx is a necessary event for sperm capacitation as well as triggering the membrane fusion in somatic cells and viruses. Many details of the potential similarities and the common components between sperm capacitation and early phases of membrane fusion have been discussed in earlier reports [52, 64]. Suffice it to say that capacitation, the physiological priming process unique to spermatozoa, like early events of membrane fusion in somatic cells and viruses, represents progressive changes and is not an all-or-nothing change [65]. 

Studies from multiple laboratories have reported that the transmembrane movement of HCO_3_^−^ anions into sperm cells may be responsible for the reported increase in intra-sperm pH during sperm capacitation [66, 67]. These ions are also reported to stimulate adenylyl cyclase, the sperm enzyme responsible for raising the levels of cAMP by increased synthesis [68, 69]. The primary target of cAMP is thought to be protein kinase A (PKA), an enzyme that is stimulated in capacitating/capacitated spermatozoa. The enzyme is a tetrameric holoenzyme complex with two regulatory (R) and two catalytic (C) subunits [70]. The complex (R2C2) displays codependent stability. The sperm PKA has a unique catalytic subunit (i.e., C*α*2). Mice lacking the catalytic subunit produce normal number of spermatozoa that swim spontaneously *in vitro*, a result consistent with the authors' conclusion that the subunit is not required for the formation of the functional flagellum. However, mice lacking the C*α*2 subunit were infertile despite normal mating behavior [70]. Also, two inhibitors of the PKA have been reported to inhibit protein tyrosine phosphorylation as well as capacitation. Taken together, these data indicate that this kinase has an important role in sperm capacitation.

To summarize the discussion on sperm capacitation, it is apparent that the loss of cholesterol is the first event that alters the permeability and fluidity of the sperm PM allowing influx of Ca^2+^ and HCO_3_^−^ ions. This influx starts a cascade of signaling events which include (a) activation of adenylyl cyclase and the production of cAMP, (b) stimulation of PKA and perhaps other kinases, and (c) protein tyrosine phosphorylation of a subset of sperm molecules. The multiple known and yet unknown biochemical changes on the sperm surface as well as inside spermatozoa are necessary before sperm cells gradually undergo capacitation. How various changes on spermatozoa fit in the puzzle that eventually initiate sperm capacitation is not yet known.

## 5. Sperm-Egg Recognition and Adhesion

As stated above, capacitated (acrosome-intact) spermatozoa interact with the zona-intact egg in a highly precise manner. Experimental evidence from multiple studies in the mouse provided evidence suggesting that binding of capacitated spermatozoa to the ZP is a two-step process. In the first step, spermatozoa loosely and reversibly adhere to the zona-intact egg by means of plasma membrane overlying the acrosome; the second step is a strong and irreversible adhesion. Many sperms can adhere to the egg surface; however, usually only one sperm will penetrate the ZP and fertilize the egg [2].

The ZP in the mouse, rat, and several other species is composed of three glycoproteins designated as ZP1, ZP2, and ZP3, and the pig has a fourth form (ZP4) as well (for review, see [2]). These glycoproteins, at least in the mouse, are synthesized and secreted by the growing oocytes during oogenesis [6]. The secreted glycoproteins interact noncovalently to form a three-dimensional network of cross-linked filaments that form the extracellular matrix. Many events of the mammalian fertilization are mediated by the matrix, including (1) relative species-specific recognition and adhesion of the opposite gametes, (2) sperm activation (i.e., induction of the AR), (3) block to polyspermy, and (4) protection of the growing embryo from fertilization to implantation [1].

In recent years, considerable progress has been made in the identification of various molecules that have some role in fertilization. In particular, work on mouse ZP (mZP) has resulted in the identification of primary (mZP3) and secondary (mZP2) binding sites for homologous spermatozoa [6]. The mZP2 and mZP3 as well as the two molecules in several other species studied are glycoproteins which show, like most glycoproteins, extensive microheterogeneity [71]. Since both glycoproteins are sulfated, the microheterogeneity and acidic nature of these glycoproteins are most likely due to N- and O-glycosylation of the polypeptide backbone and sulfation of the glycan chains.

Several lines of evidence listed in previous review articles strongly suggest that mammalian fertilization is a carbohydrate-mediated event (for reviews see [2, 45]). Experimental details included in these articles agree with the conclusion that mZP3 (but not mZP2) is recognized by the capacitated and acrosome-intact spermatozoa. Furthermore, the experimental evidence listed in the Wassarman's article [45] provided evidence suggesting that glycan portion of mZP3 glycoprotein is the ligand for sperm surface receptor(s). Despite numerous advances, considerable controversy remains regarding the precise identity of the terminal sugar residue(s) responsible for the ligand activity of the mZP3. For instance, studies published in the 1980s suggested that the sperm-binding activity was associated with the *α*-linked galactosyl residue(s) present at the non-reducing terminus of an O-linked oligosaccharide [72] or with the N-acetylglucosaminyl residue(s) [11, 73]. In the 1990s, additional sugar residues including mannosyl [8, 9], N-acetylgalactosaminyl [74], sialyl [10], and fucosyl [13] were added to the list of sugars thought to mediate sperm-egg binding.

In the late 1990s, investigators used gene disruption approaches to address the role of *α*1,3-galactose or N-acetylglucosamine in fertilization. Female *α*-1,3 GT (−/−) mice yielded oocytes that were devoid of *α*-1,3 Gal-epitopes. However, these mice were fully fertile, a result consistent with the investigators' conclusion that *α*-1,3 Gal-epitopes are not required for fertilization in the mouse [75]. Similarly, mice devoid of sperm *β*-1,4-galactosyltransferase, an enzyme thought to recognize and bind to N-acetylglucosaminyl residue(s) on mZP3, were still fertile [76]. Taken together, these data provide evidence indicating that *α*-1,3 Gal-epitopes on mZP and sperm surface galactosyltransferase are not required for fertilization in the mouse. To the best of our knowledge, there are no genetically engineered mouse models to rule out the suggested role of mannose, galactosamine, sialic acid, or *α*-L-fucose residue(s) in sperm-egg adhesion.

As stated above, mZP3 is highly glycosylated containing a variety of N-linked glycan chains including high mannose/hybrid-type, bi-, tri-, and tetra-antennary complex-type, and poly-N-acetyllactosaminyl type in addition to an O-linked trisaccharide [71]. Over 20 years ago, our group demonstrated the presence of high mannose/hybrid-type glycans on mZP2 and mZP3 [77]. We also presented evidence suggesting that these glycan chains may be a part of the recognition/binding site(s) for sperm PM mannosidase [78]. The enzyme is a glycosidase and belongs to a class of enzymes responsible for hydrolytic cleavage of glycosidic bonds. The catalytic mechanism of action of these enzymes is thought to follow the model advanced for lysozyme. In such a model, there are two important carboxylic acid moieties in the active site: one ionized and the other protonated [79]. The former moiety stabilizes the resulting oxycarbonium ion, either by ion pair interaction or by covalent bonding, whereas the latter moiety facilitates departure of the cleaving group. This implies that all hydrolytic enzymes have a common catalytic mechanism. The common feature of this mechanism is the formation of an enzyme: substrate (sugar) intermediate before cleavage of the sugar residue. Because of this interaction between the enzyme and substrate, it has been proposed that cell surface glycosidases have a role in cell-cell adhesion [80]. Since purified *α*-D-mannosidase cleaves negligible amounts of [^3^H] mannosyl residues from [^3^H] mannose-containing glycoproteins after 4 h of incubation, it is surmised that an intermediate complex of sperm enzyme (*α*-D-mannosidase) and zona substrate (high mannose/hybrid-type glycan chains) is formed that leads to the next step in fertilization.

It is interesting that like mZP3, the porcine ZP glycoprotein 3 (pZP3) has been reported to possess sperm receptor activity. The 55 kDa pZP3 is also highly glycosylated containing N- and O-linked glycan units as well as poly-N-acetyllactosaminyl glycans [81]. Structural analysis of the N-linked glycan chains on pZP3 shows the presence of neutral and acidic chains. However, only the neutral N-linked glycan chains were demonstrated to inhibit sperm-egg binding *in vitro*, a result consistent with the suggestion that N-linked neutral glycan chains have a role in sperm recognition and adhesion [82, 83]. 

Human spermatozoa have been reported to contain *α*-D-mannosidase [78] and a mannose-binding protein [84]. Each of these macromolecules has been proposed to have a receptor-like role in binding to mannose-containing oligosaccharide(s) on the ZP3 [9]. It has also been suggested that a human sperm antigen designated FA-1 [85] and a selectin-like molecule [86] bind to homologous ZP. Several other sperm surface macromolecules in various species have been proposed to function as receptor molecules. These include: (a) a trypsin-inhibitor sensitive site [87], (b) a ZP3-binding 56-kDa sperm protein [88], (c) a 95-kDa sperm protein [89] that has been suggested to be a unique hexokinase [90], (d) zonadhesin, a molecule suggested to be essential for species specificity of sperm adhesion to the ZP [91]; (e) a fucose-binding protein that recognizes carbohydrate moiety on ZP glycoprotein [92]; (f) sperm protein 38 [93]; (g) hyaluronidase [94]; and (h) a trypsin-like serine protease [95]. Recent evidence suggests that spermatozoa without hyaluronidase (PH-20) or trypsin-like proteolytic enzyme, acrosin, are still fertile [87]. These results suggest that, at least in the mouse, PH-20 and acrosin are not essential for fertilization.

This brief summary of the research from numerous investigators suggests that a carbohydrate recognition mechanism is involved in sperm-zona (egg) recognition and adhesion. Despite the overwhelming evidence in favor of glycans of ZP being ligand(s) for the sperm surface receptor(s), a recent publication by Dean and associates presented evidence suggesting that the gamete recognition in mice depends on the cleavage status of a ZP2 protein [14]. The investigators argue that a ZP2 cleavage model for the recognition of the opposite gametes requires an intact ZP2, whereas a glycan release model postulates that the ZP glycan residues are ligands for spermatozoa. These two models were tested by replacing endogenous ZP2 with a mutant ZP2 that cannot be cleaved and a glycan release model where ZP3 lacks O-linked glycan(s). Data presented demonstrate that spermatozoa bound to the two-cell ZP2 mutant embryos despite fertilization and cortical granule exocytosis. However, despite the absence of the O-linked glycan residues from ZP3 mutant eggs, spermatozoa still fertilized them. These data, according to investigators, demonstrate that sperm-egg recognition depends on the cleavage status of ZP2 [14].

The investigators of the above studies, however, did not address three important points. First, the structural similarities/dissimilarities of the endogenous and the mutant ZP2. Second, has the replacement of endogenous ZP2 glycoprotein with mutant ZP2 protein altered the three-dimensional structure of the egg coat? Finally, the possible outcome if all or some of the other sugar residues implicated in sperm-egg recognition (see above) were removed from the ZP3. Some similar concerns were also raised in a recent article [96]. Unless these concerns have been satisfactorily addressed, the final decision for the cause of sperm-egg recognition and adhesion still remains in the hands of jurors. From the above discussion, it is reasonable to conclude that the mechanism(s) underlying interaction of the opposite gametes still remains an unsolved issue.

## 6. The Sperm Acrosome and Exocytosis of Acrosomal Contents

The sperm acrosome plays an important role following species-specific sperm-egg (zona) binding. Clinical studies have identified a group of men whose infertility is associated with abnormal AR [84]. Thus, a brief discussion of the sperm acrosome and its organization is provided to better understand its role in fertilization. A well-developed acrosome is a sac-like structure with an inner (IAM) and an outer acrosomal membrane (OAM) that covers the anterior portion of the nucleus. The acrosome acts in concert with the plasma membrane overlying the OAM. The size and shape of the acrosome varies from species to species and depends on the morphology of the sperm head. It generally falls into two categories, sickle-shaped in rodents and skull-cap/ paddle-shaped in several larger animals. It is a Golgi-derived secretory organelle that resembles the cellular lysosome in many ways; however, the acrosome is considered analogous to a secretory organelle. It undergoes secretion (exocytosis) as a result of an external stimulus [46].

In the mouse and several other species, the signal that initiates the AR is thought to be the recognition and irreversible binding of capacitated spermatozoa to the ZP glycoprotein 3 in a receptor-ligand manner (see above). The protein backbone of the glycoprotein 3 (mZP3) facilitates the aggregation of the sperm surface receptor(s) prior to induction of the AR [73]. Indeed, our group has demonstrated that specific sugar residues (mannose, N-acetylglucosamine, and N-acetylgalactosamine) can induce the acrosome reaction only when they are linked to a protein backbone as in neoglycoproteins [74].

Morphologically, the AR occurs in several steps. First, the irreversible binding of opposite gametes causes a sustained increase in Ca^2+^ between the sperm PM and the OAM [46]. Second, there is a gradual fusion of these two membranes at multiple sites in the anterior head region. Third, as the PM and the OAM fuse, there is a formation of hybrid membranes (hybrid vesicles) and the release of acrosomal contents in a time-dependent manner. The entire process is slow and may be regulated by cytoskeletal elements present between the sperm PM and the OAM. The fusion of these membranes causes the release of acrosomal contents containing powerful enzymes at the site of sperm-zona binding. The action of these powerful enzymes makes it possible for the hyperactive spermatozoon to penetrate the ZP and fertilize the egg [3]. 

It should be noted that the levels of Ca^2+^ in the sperm cells are low before their binding to the zona-intact egg, whereas the concentration of the divalent cation is much higher in the extracellular fluid. The irreversible binding of sperm and egg activates Ca^2+^ channels that cause a sustained increase in the divalent cation and other second messengers, such as cAMP and IP3. The increase in cAMP causes activation of protein kinases and phospholipid-dependent kinases. The transient rise in intrasperm Ca^2+^ and other second messengers also starts a cascade of signaling events that elevate intrasperm pH and triggers the fusion of the sperm PM and the OAM at multiple sites and release of the acrosomal contents [2, 46]. 

Does Ca^2+^-triggered AR use SNARE and other molecules that take part in Ca^2+^-regulated membrane fusion in somatic cells and viruses? As discussed in our earlier article [64], sperm capacitation, a process unique to the male gamete, is a result of the membrane priming which is likely analogous to early phases of membrane fusion during secretory and endocytotic pathways among eukaryotes and viruses. Thus it seems likely that following the assembly of fusion machinery during sperm capacitation, their fusion during the AR will also use various molecules that are involved in the fusion of somatic cell membranes and viruses. The potential involvement of several common components and striking similarities in these two events allow us to suggest that the AR is likely analogous to the final phase of the membrane fusion in somatic cells and viruses.

In addition to the ZP which is considered a primary inducer of the AR, there is a long list of physiological and nonphysiological compounds that are known to induce the AR in capacitated spermatozoa (for review, see [46]). The physiological inducers of the AR include progesterone, prostaglandins, sterol sulphate, glycosaminoglycans, the epididymal growth factor, atriopeptin, platelet activating factor, and ATP. In addition to the physiological inducers of the AR, there is a long list of nonphysiological compounds that induce the AR. These include calcium ionophore, lectins, neoglycoproteins, and many more [3, 74]. 

### 6.1. Molecular Mechanisms That Regulate the Acrosomal Exocytosis

The interaction of sperm and egg triggers the signaling pathway that activates spermatozoa by opening of Ca^2+^ channels on the sperm PM. This step elevates levels of intrasperm Ca^2+^ and other second messengers through appropriate transducers and effectors which initiate a cascade of signaling events that result in the AR and exocytosis of the acrosomal contents. These contents include glycohydrolases, proteinases, esterases, phosphatases, phospholipases, sulphatases, and so forth. as described [3]. Several possible mechanisms have been discussed in previous reports and our recent review article [46]. Suffice it to say that Ca^2+^ plays a central role in the assembly of fusion machinery during capacitation and subsequent fusion of the sperm PM and the OAM during acrosomal exocytosis. The Ca^2+^ entry is regulated by several transport channels including exchanger channel, voltage-sensitive/-insensitive channels and inositol triphosphate (IP3)-gated channels. Combined, these channels regulate the flow of Ca^2+^ and other ions and form an early component of the signal transduction pathway. However, to the best of our knowledge, there is no unified view as yet that can explain the elevation in intrasperm pH. 

It should be noted that phospholipases A2 and C are activated by Ca^2+^ and are believed to have a role in the acrosomal exocytosis. Many details of how the activation of phospholipases regulate the AR have been discussed in an earlier article [3]. In addition to phospholipases, there is a long list of sperm molecules suggested to have a role in induction of the AR. These include (i) a 95 kDa sperm PM molecule which is phosphorylated as a consequence of its aggregation by ZP3, (ii) protein tyrosine kinases, a family of intrinsic signal transduction receptors which undergo protein tyrosine phosphorylation in response to sperm-egg interaction, (iii) calmodulin, a 17 kDa acidic Ca^2+^-binding protein present in the sperm head and flagellum, regulates many Ca^2+^ signaling pathways and membrane fusion events in spermatozoa and somatic cells by modulating the activity of enzymes and ion pumps. Mammalian spermatozoa also contain a calmodulin acceptor protein, synaptical vesicle protein, Rab3a/A, a small GTPase, G-proteins, soluble N-ethylmaleimide attachment protein receptor (SNARE) molecule, and angiotensin ll receptor that have been suggested to have a regulatory role in triggering the AR [46]. Other protein and nonprotein components that have been suggested to have a role in triggering the AR can be found in a book chapter [97]. It is not yet known whether various sperm molecules trigger the membrane fusion events individually or as a multiple complex.

In the recent years it has become apparent that all living cells, including spermatozoa, receive extracellular and intracellular signals and translate them for normal functioning. In this section, we have attempted to highlight the biological processes thought to be important in sperm function and fertilization. 

## 7. Secondary Binding Events and Sperm-Egg Fusion

The fusion of the sperm PM and the OAM at multiple sites allows the acrosomal contents to escape (see above), thus exposing the inner acrosomal membrane. The exposed membrane contains a new set of binding sites specific for the mZP2 [1]. These binding sites hold the acrosome-reacted (hyperactive) sperm and zona-intact egg in contact before the acrosome-reacted spermatozoon penetrates the zona relying on the hydrolytic acrosomal enzymes and the enhanced thrust generated by the hyperactivated beat pattern of the bound spermatozoon [1, 2]. Although the molecules involved in the secondary binding and sperm-egg fusion events will be of interest to many readers of this paper, they are beyond the scope of this paper. Interested readers are referred to the following interesting and informative article [98]. 

## 8. Conclusions

This paper covers many aspects of mammalian sperm-egg recognition and adhesion that lead to fertilization. It is apparent from the discussion in the paper that extensive progress has been made in this field of reproductive biology and physiology. We have attempted to highlight molecular processes thought to be important in sperm function and fertilization. Although the molecule(s) that initiates sperm-egg recognition and adhesion remains controversial, it is a matter of time before this controversy is settled. It is important to keep in mind that many advances in the field of *in vitro* fertilization (IVF) procedures and intracytoplasmic sperm injection (ICSI) protocols appear to sideline the importance of interacting molecules on opposite gametes. However, the significance of these molecules should not be undermined during *in vivo* fertilization. An understanding of the function of various sperm molecules in the process of *in vivo* fertilization will allow new strategies to regulate these events and alter sperm function and male fertility.

## Abbreviations

AR: Acrosome reaction
IAM: Inner acrosomal membrane
OAM: Outer acrosomal membrane
PM: Plasma membrane
IVF: *In vitro* fertilization
ICSI: Intracytoplasmic sperm injection
ZP: Zona pellucida.

## References

[1] Yanagimachi R, Knobil E, Neil JD (1994). Mammalian fertilization. *Physiology of Reproduction*.

[2] Tulsiani DRP, Yoshida-Komiya H, Araki Y (1997). Mammalian fertilization: a carbohydrate-mediated event. *Biology of Reproduction*.

[3] Tulsiani DRP, Abou-Haila A, Loeser CR, Pereira BMJ (1998). The biological and functional significance of the sperm acrosome and acrosomal enzymes in mammalian fertilization. *Experimental Cell Research*.

[4] Tulsiani DRP, Abou-Haila A (2001). Mammalian sperm molecules that are potentially important in interaction with female genital tract and egg vestments. *Zygote*.

[5] Tulsiani DRP, Abou-Haila A (2011). Molecular events that regulate mammalian fertilization. *Minerva Ginecologica*.

[6] Wassarman PM (1988). Zona pellucida glycoproteins. *Annual Review of Biochemistry*.

[7] Shalgi R, Matityahu A, Nebel L (1986). The role of carbohydrates in sperm-egg interaction in rats. *Biology of Reproduction*.

[8] Cornwall GA, Tulsiani DRP, Orgebin-Crist MC (1991). Inhibition of the mouse sperm surface *α*-D-mannosidase inhibits sperm-egg binding *in vitro*. *Biology of Reproduction*.

[9] Yoshida-Komiya H, Tulsiani DRP, Hirayama T, Araki Y (1999). Mannose-binding molecules of rat spermatozoa and sperm-egg interaction. *Zygote*.

[10] Lambert H (1984). Role of sperm-surface glycoproteins in gamete recognition in two mouse species. *Journal of Reproduction and Fertility*.

[11] Shur BD, Shatten H, Schatten G (1989). Galactosyltransferase as a recognition molecule during fertilization and development. *Molecular Biology of Fertilization*.

[12] Florman HM, Bechtol KB, Wassarman PM (1984). Enzymatic dissection of the functions of the mouse egg’s receptor for sperm. *Developmental Biology*.

[13] Johnston DS, Wright WW, Shaper JH, Hokke CH, van den Eijnden DH, Joziasse DH (1998). Murine sperm-zona binding, a fucosyl residue is required for a high affinity sperm-binding ligand : a second site on sperm binds a nonfucosylated, *β*-galactosyl-capped oligosaccharide. *Journal of Biological Chemistry*.

[14] Gahlay G, Gauthier L, Baibakov B, Epifano O, Dean J (2010). Gamete recognition in mice depends on the cleavage status of an egg’s zona pellucida protein. *Science*.

[15] Clermont Y, Oko R, Hermo L, Desjardins C, Ewing J (1993). Cell biology of mammalion spermatogenesis. *Cell and Molecular Biology of the Testis*.

[16] Eddy F, O'Brien D, Knobil SE, Neil J (1994). The spermatozoon. *Physiology of Reproduction*.

[17] Abou-Haila A, Tulsiani DRP (2000). Mammalian sperm acrosome: formation, contents, and function. *Archives of Biochemistry and Biophysics*.

[18] Hecht NB (1998). Molecular mechanisms of male germ cell differentiation. *BioEssays*.

[19] Gerton GL, Millette CF (1986). Stage-specific synthesis and fucosylation of plasma membrane proteins by mouse pachytene spermatocytes and round spermatids in culture. *Biology of Reproduction*.

[20] Neurath H (1989). Proteolytic processing and physiological regulation. *Trends in Biochemical Sciences*.

[21] Blobel CP, Myles DG, Primakoff P, White JM (1990). Proteolytic processing of a protein involved in sperm-egg fusion correlates with acquisition of fertilization competence. *Journal of Cell Biology*.

[22] Orgebin-Crist M-C, Kretzer DM, Spera G (1987). Morphological basis of human reproduction. *Acta Medica*.

[23] Jones R (1998). Plasma membrane structure and remodelling during sperm maturation in the epididymis. *Journal of Reproduction and Fertility. Supplement*.

[24] Dacheux JL, Gatti JL, Dacheux F (2003). Contribution of epididymal secretory proteins for spermatozoa maturation. *Microscopy Research and Technique*.

[25] Toshimori K (2003). Biology of spermatozoa maturation: an overview with an introduction to this issue. *Microscopy Research and Technique*.

[26] Tulsiani DRP (2006). Glycan-modifying enzymes in luminal fluid of the mammalian epididymis: an overview of their potential role in sperm maturation. *Molecular and Cellular Endocrinology*.

[27] Srivastava A, Olson GE (1991). Glycoprotein changes in the rat sperm plasma membrane during maturation in the epididymis. *Molecular Reproduction and Development*.

[28] Skudlarek MD, Tulsiani DRP, Nagdas SK, Orgebin-Crist MC (1993). *β*-D-galactosidase of rat spermatozoa: subcellular distribution, substrate specificity, and molecular changes during epididymal maturation. *Biology of Reproduction*.

[29] Skudlarek MD, Tulsiani DRP, Orgebin-Crist M-C (1992). Rat epididymal luminal fluid acid *β*-D-galactosidase optimally hydrolyses glycoprotein substrate at neutral pH. *Biochemical Journal*.

[30] Tulsiani DRP, Skudlarek MD, Araki Y, Orgebin-Crist MC (1995). Purification and characterization of two forms of *β*-D-galactosidase from rat epididymal luminal fluid: evidence for their role in the modification of sperm plasma membrane glycoprotein(s). *Biochemical Journal*.

[31] Tulsiani DRP, Skudlarek MD, Holland MK, Orgebin-Crist MC (1993). Glycosylation of rat sperm plasma membrane during epididymal maturation. *Biology of Reproduction*.

[32] Tulsiani DR, Orgebin-Crist MC, Skudlarek MD (1998). Role of luminal fluid glycosyltransferases and glycosidases in the modification of rat sperm plasma membrane glycoproteins during epididymal maturation. *Journal of Reproduction and Fertility. Supplement*.

[33] Dias Alves MS, Martins MS, Pena SD (1995). Monoclonal antibody against a 52 K sperm surface protein inhibits sperm-zona pellucida interactions in the rat. *Journal of Experimental Zoology*.

[34] Toshimori K (1998). Maturation of mammalian spermatozoa: modifications of the acrosome and plasma membrane leading to fertilization. *Cell and Tissue Research*.

[35] Skudlarek MD, Abou-Haila A, Tulsiani DRP (2000). Rat spermatogenic cell *β*-D-galactosidase: characterization, biosynthesis, and immunolocalization. *Experimental Cell Research*.

[36] Hancock LW, Raab LS, Aronson NN (1993). Synthesis and processing of rat sperm-associated *α*-L-fucosidase. *Biology of Reproduction*.

[37] Nagdas SK, Skudlarek MD, Orgebin-Crist M-C, Tulsiani DRP (1992). Biochemical alterations in the proacrosin-acrosin system during epididymal maturation of the rat spermatozoa. *Journal of Andrology*.

[38] Kurth BE, Wright RM, Flickinger CJ, Herr JC (1993). Stage-specific detection of mRNA for the sperm antigen SP-10 in human testes. *Anatomical Record*.

[39] Anakwe OO, Gerton GL (1990). Acrosome biogenesis begins during meiosis: evidence from the synthesis and distribution of an acrosomal gylcoprotein, acrogranin, during guinea pig spermatogenesis. *Biology of Reproduction*.

[40] Bozzola JJ, Polakoski K, Haas N, Russell LD, Campbell P, Peterson RN (1991). Localization of boar sperm proacrosin during spermatogenesis and during sperm maturation in the epididymis. *American Journal of Anatomy*.

[41] Mukerji SK, Meizel S (1979). Rabbit testis proacrosin. Purification, molecular weight estimation, and amino acid and carbohydrate composition of the molecule. *Journal of Biological Chemistry*.

[42] Baba T, Kashiwabara SI, Watanabe K (1989). Activation and maturation mechanisms of boar acrosin zymogen based on the deduced primary structure. *Journal of Biological Chemistry*.

[43] Austin CR (1951). Observations on the penetration of the sperm in the mammalian egg. *Australian Journal of Scientific Research B*.

[44] Chang MC (1951). Fertilizing capacity of spermatozoa deposited into the fallopian tubes. *Nature*.

[45] Wassarman PM (1987). Early events in mammalian fertilization. *Annual Review of Cell Biology*.

[46] Abou-haila A, Tulsiani DRP (2009). Signal transduction pathways that regulate sperm capacitation and the acrosome reaction. *Archives of Biochemistry and Biophysics*.

[47] Davis BK, Byrne R, Hungund B (1979). Studies on the mechanism of capacitation. II. Evidence for lipid transfer between plasma membrane of rat sperm and serum albumin during capacitation *in vitro*. *Biochimica et Biophysica Acta*.

[48] Dow MPD, Bavister BD (1989). Direct contact is required between serum albumin and hamster spermatozoa for capacitation *in vitro*. *Gamete Research*.

[49] Zeng HT, Tulsiani DRP (2003). Calmodulin antagonists differentially affect capacitation-associated protein tyrosine phosphorylation of mouse sperm components. *Journal of Cell Science*.

[50] Cross NL (1998). Role of cholesterol in sperm capacitation. *Biology of Reproduction*.

[51] Ahuja KK (1984). Lectin-coated agarose beads in the investigation of sperm capacitation in the hamster. *Developmental Biology*.

[52] Abou-Haila A, Tulsiani DRP (2003). Evidence for the capacitation-associated membrane priming of mouse spermatozoa. *Histochemistry and Cell Biology*.

[53] Yanagimachi R, Chang MC (1963). Sperm ascent through the oviduct of the hamster and rabbit in relation to the time of ovulation. *Journal of Reproduction and Fertility*.

[54] Olds-Clarke P, Bavister B, Cummins J, Rolden ERS (1990). Variation in the quality of sperm motility and its relationship to capacitation. *Fertilization in Mammals*.

[55] Visconti PE, Bailey JL, Moore GD, Olds-Clarke P, Kopf GS (1995). Capacitation of mouse spermatozoa. 1. Correlation between the capacitation state and protein tyrosine phosphorylation. *Development*.

[56] Visconti PE, Kopf GS (1998). Regulation of protein phosphorylation during sperm capacitation. *Biology of Reproduction*.

[57] Mandal A, Naaby-Hansen S, Wolkowicz MJ (1999). FSP95, a testis-specific 95-kilodalton fibrous sheath antigen that undergoes tyrosine phosphorylation in capacitated human spermatozoa. *Biology of Reproduction*.

[58] Westbrook VA, Diekman AB, Herr JC, Visconti PE, Tulsiani D (2003). Capacitation: signaling pathways involved in sperm acquisition of fertilization capacity. *Introduction to Mammalian Fertilization*.

[59] Marín-Briggiler CI, Jha KN, Chertihin O (2005). Evidence of the presence of calcium/calmodulin-dependent protein kinase IV in human sperm and its involvement in motility regulation. *Journal of Cell Science*.

[60] Cheung WY (1980). Calmodulin plays a pivotal role in cellular regulation. *Science*.

[61] Means AR, Lagace L, Guerriero V, Chafouleas JG (1982). Calmodulin as a mediator of hormone action and cell regulation. *Journal of Cellular Biochemistry*.

[62] Kann ML, Feinberg J, Rainteau D, Dadoune JP, Weinman S, Fouquet JP (1991). Localization of Calmodulin in perinuclear structures of spermatids and spermatozoa: a comparison of six mammalian species. *Anatomical Record*.

[63] Bendahmane M, Lynch C, Tulsiani DRP (2001). Calmodulin signals capacitation and triggers the agonist-induced acrosome reaction in mouse spermatozoa. *Archives of Biochemistry and Biophysics*.

[64] Tulsiani DRP, Abou-Haila A (2004). Is sperm capacitation analogous to early phases of Ca^2+^-triggered membrane fusion in somatic cells and viruses?. *BioEssays*.

[65] Kim KS, Cha MC, Gerton GL (2001). Mouse sperm protein sp56 is a component of the acrosomal matrix. *Biology of Reproduction*.

[66] Uguz C, Vredenburgh WL, Parrish JJ (1994). Heparin-induced capacitation but not intracellular alkalinization of bovine sperm is inhibited by Rp-adenosine-3’,5’-cyclic monophosphorothioate. *Biology of Reproduction*.

[67] Zeng Y, Oberdorf JA, Florman HM (1996). pH regulation in mouse sperm: identification of Na^+^-, Cl^−^-, and HCO^−3^-dependent and arylaminobenzoate-dependent regulatory mechanisms and characterization of their roles in sperm capacitation. *Developmental Biology*.

[68] Okamura N, Tajima Y, Soejima A (1985). Sodium bicarbonate in seminal plasma stimulates the motility of mammalian spermatozoa through direct activation of adenylate cyclase. *Journal of Biological Chemistry*.

[69] Visconti PE, Muschietti JP, Flawia MM, Tezon JG (1990). Bicarbonate dependence of cAMP accumulation induced by phorbol esters in hamster spermatozoa. *Biochimica et Biophysica Acta*.

[70] Nolan MA, Babcock DF, Wennemuth G, Brown W, Burton KA, McKnight GS (2004). Sperm-specific protein kinase A catalytic subunit C*α*2 orchestrates cAMP signaling for male fertility. *Proceedings of the National Academy of Sciences of the United States of America*.

[71] Tulsiani DRP (2000). Structural analysis of the asparagine-linked glycan units of the ZP2 and ZP3 glycoproteins from mouse zona pellucida. *Archives of Biochemistry and Biophysics*.

[72] Bleil JD, Wassarman PM (1988). Galactose at the nonreducing terminus of O-linked oligosaccharides of mouse egg zona pellucida glycoprotein ZP3 is essential for the glycoprotein’s sperm receptor activity. *Proceedings of the National Academy of Sciences of the United States of America*.

[73] Shur BD (1998). Is sperm galactosyltransferase a signaling subunit of a multimeric gamete receptor?. *Biochemical and Biophysical Research Communications*.

[74] Loeser CR, Tulsiani DRP (1999). The role of carbohydrates in the induction of the acrosome reaction in mouse spermatozoa. *Biology of Reproduction*.

[75] Thall AD, Maly P, Lowe JB (1995). Oocyte Gal*α*1,3Gal epitopes implicated in sperm adhesion to the zona pellucida glycoprotein ZP3 are not required for fertilization in the mouse. *Journal of Biological Chemistry*.

[76] Williams SA, Xia L, Cummings RD, McEver RP, Stanley P (2007). Fertilization in mouse does not require terminal galactose or N-acetylglucosamine on the zona pellucida glycans. *Journal of Cell Science*.

[77] Tulsiani DRP, Nagdas SK, Cornwall GA, Orgebin-Crist MC (1992). Evidence for the presence of high-mannose/hybrid oligosaccharide chain(s) on the mouse ZP2 and ZP3. *Biology of Reproduction*.

[78] Tulsiani DRP, Skudlarek MD, Orgebin-Crist MC (1989). Novel *α*-D-mannosidase of rat sperm plasma membranes: characterization and potential role in sperm-egg interactions. *Journal of Cell Biology*.

[79] Ichikawa Y, Look GC, Wong CH (1992). Enzyme-catalyzed oligosaccharide synthesis. *Analytical Biochemistry*.

[80] Rauvala H, Hakomori SI (1981). Studies on cell adhesion and recognition. III. The occurrence of *α*-mannosidase at the fibroblast cell surface, and its possible role in cell recognition. *Journal of Cell Biology*.

[81] Yurewicz EC, Sacco AG, Subramanian MG (1987). Structural characterization of the M(r) = 55,000 antigen (ZP3) of porcine oocyte zona pellucida. Purification and characterization of *α*- and *β*-glycoproteins following digestion of lactosaminoglycan with endo-*β*-galactosidase. *Journal of Biological Chemistry*.

[82] Noguchi S, Hatanaka Y, Tobita T, Nakano M (1992). Structural analysis of the N-linked carbohydrate chains of the 55-kDa glycoprotein family (PZP3) from porcine zona pellucida. *European Journal of Biochemistry*.

[83] Yonezawa N, Aoki H, Hatanaka Y, Nakano M (1995). Involvement of N-linked carbohydrate chains of pig zona pellucida in sperm-egg binding. *European Journal of Biochemistry*.

[84] Benoff S (1997). Carbohydrates and fertilization: an overview. *Molecular Human Reproduction*.

[85] Naz RK, Phillips TM, Rosenblum BB (1986). Characterization of the fertilization antigen 1 for the development of a contraceptive vaccine. *Proceedings of the National Academy of Sciences of the United States of America*.

[86] Dell A, Morris HR, Easton RL (1995). Structural analysis of the oligosaccharides derived from glycodelin, a human glycoprotein with potent immunosuppressive and contraceptive activities. *Journal of Biological Chemistry*.

[87] Baba T, Azuma S, Kashiwabara SI, Toyoda Y (1994). Sperm from mice carrying a targeted mutation of the acrosin gene can penetrate the oocyte zona pellucida and effect fertilization. *Journal of Biological Chemistry*.

[88] Cheng A, Le T, Palacios M (1994). Sperm-egg recognition in the mouse: characterization of sp56, a sperm protein having specific affinity for ZP3. *Journal of Cell Biology*.

[89] Leyton L, Saling P (1989). 95 kd sperm protein binds ZP3 and serve as tyrosine kinase substrates in response to zona binding. *Cell*.

[90] Kalab P, Visconti P, Leclerc P, Kopf GS (1994). p95, the major phosphotyrosine-containing protein in mouse spermatozoa, is a hexokinase with unique properties. *Journal of Biological Chemistry*.

[91] Hardy DM, Garbers DL (1995). A sperm membrane protein that binds in a species-specific manner to the egg extracellular matrix is homologous to von Willebrand factor. *Journal of Biological Chemistry*.

[92] Huang TTF, Ohzu E, Yanagimachi R (1982). Evidence suggesting that L-fucose is part of a recognition signal for sperm-zona pellucida attachment in mammals. *Gamete Research*.

[93] Mori E, Baba T, Iwamatsu A, Mori T (1993). Purification and characterization of a 38-kDa protein, sp38, with zona pellucida-binding property from porcine epididymal sperm. *Biochemical and Biophysical Research Communications*.

[94] Lin Y, Mahan K, Lathrop WF, Myles DG, Primakoff P (1994). A hyaluronidase activity of the sperm plasma membrane protein PH-20 enables sperm to penetrate the cumulus cell layer surrounding the egg. *Journal of Cell Biology*.

[95] Jones R, Milligan S (1988). Membrane remodeling during sperm maturation in the epididymis. *,Oxford Reviews of Reproductive Biology*.

[96] Visconti PE, Florman HM (2010). Mechanisms of sperm-egg interactions: between sugars and broken bonds. *Science Signaling*.

[97] Tulsiani DRP, Abou-Haila A, Tulsiani D (2003). Sperm-egg interaction and exocytosis of
acrosomal contents. *Introduction to Mammalian Fertilization*.

[98] McLeskey SB, Dowds C, Carballada R, White RR, Saling PM (1998). Molecules involved in mammalian sperm-egg interaction. *International Review of Cytology*.

